# Coronary microvascular dysfunction in Takotsubo syndrome: an analysis using angiography-derived index of microcirculatory resistance

**DOI:** 10.1007/s00392-023-02329-7

**Published:** 2023-11-20

**Authors:** Victor Schweiger, Thomas Gilhofer, Rick Fang, Alessandro Candreva, Burkhardt Seifert, Davide Di Vece, Michael Wuerdinger, Iva Koleva, Katja Rajman, Maciej Cieslik, Alexander Gotschy, Jonathan Michel, Julia Stehli, David Niederseer, Linn Ryberg, Jelena Ghadri, Frank Ruschitzka, Barbara Stähli, Victoria Lucia Cammann, Christian Templin

**Affiliations:** 1 Department of Cardiology, University Heart Centre, University Hospital Zurich, University of Zurich, Raemistrasse 100, 8091 Zurich, Switzerland; 2 Suzhou Rainmed Medical Technology Co., Ltd, Building 31, Northeast District, Nano City, No. 99 Jinji Lake Avenue, Suzhou Industrial Park, Suzhou, China; 3 Division of Biostatistics, Epidemiology, Biostatistics, and Prevention Institute, University of Zurich, Raemistrasse 100, 8091 Zurich, Switzerland

**Keywords:** Angiography-derived IMR, Index of microcirculatory resistance, Takotsubo syndrome, Coronary microvascular dysfunction

## Abstract

**Background:**

Coronary microvascular dysfunction (CMD) has been proposed as a crucial factor in the pathophysiology of Takotsubo syndrome (TTS). The angiography-derived index of microcirculatory resistance (caIMR) offers an alternative to conventional hyperemic wire-based IMR to assess CMD. We aimed to evaluate CMD’s prevalence, transience, and impact on in-hospital outcomes in TTS.

**Methods:**

All three coronary arteries of 96 patients with TTS were assessed for their coronary angiography derived Index of microcirculatory Resistance (caIMR) and compared to non-obstructed vessels of matched patients with ST-elevation myocardial infarction. Further, the association between caIMR and the TTS-specific combined in-hospital endpoint of death, cardiac arrest, ventricular arrhythmogenic events and cardiogenic shock was investigated.

**Results:**

Elevated IMR was present in all TTS patients, with significantly elevated caIMR values in all coronary arteries compared to controls. CaIMR did not differ between apical and midventricular TTS types. CaIMR normalized in TTS patients with follow-up angiographies performed at a median of 28 months (median caIMR at event vs follow-up: LAD 34.8 [29.9–41.1] vs 20.3 [16.0–25.3], *p* < 0.001; LCX: 38.7 [32.9–50.1] vs 23.7 [19.4–30.5], *p* < 0.001; RCA: 31.7 [25.0–39.1] vs 19.6 [17.1–24.0], *p* < 0.001). The extent of caIMR elevation significantly correlated with the combined in-hospital endpoint (*p* = 0.036).

**Conclusion:**

TTS patients had evidence of elevated caIMR in at least one coronary artery with a trend towards higher LAD caIMR in apical type TTS and normalization after recovery. Furthermore, extent of caIMR elevation was associated with increased risk of in-hospital MACE of TTS patients.

**Graphical abstract:**

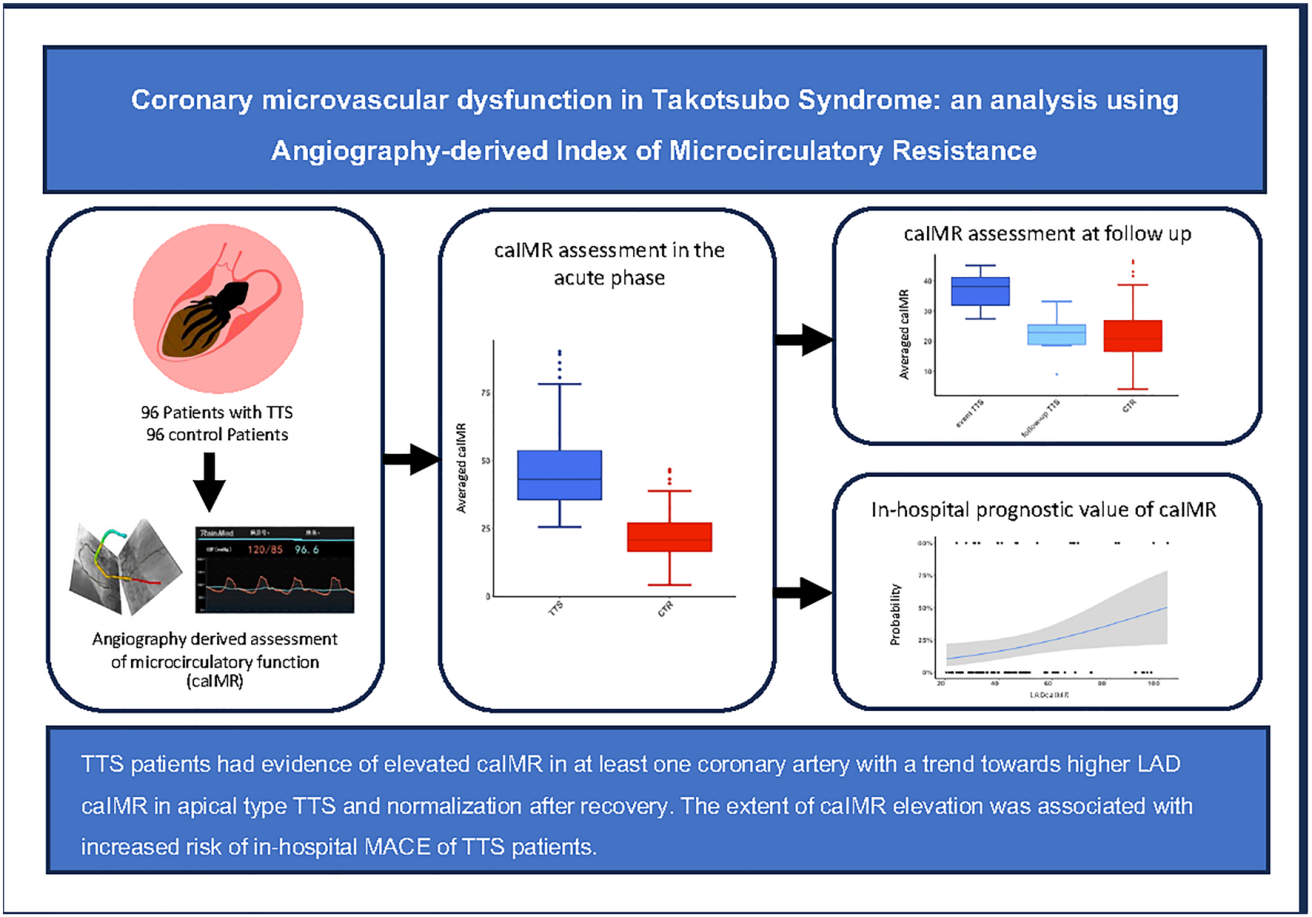

**Supplementary Information:**

The online version contains supplementary material available at 10.1007/s00392-023-02329-7.

## Introduction

Takotsubo syndrome (TTS) is an acute heart failure syndrome with substantial morbidity and mortality characterized by specific transient ventricular wall motion abnormalities [1–3]. The precise pathophysiological mechanisms are still a matter of debate and remain incompletely understood [4].

Coronary microvascular dysfunction has been widely proposed as a significant contributing mechanism in the pathophysiological development of TTS [5]. The assessment and quantification of CMD can be achieved through the index of microcirculatory resistance (IMR), an invasive, wire-based method that evaluates coronary microvascular function in both resting and hyperemic states induced by intravenous Adenosine [6–8].

The newly developed angiography-based indices of microcirculatory resistance derive from the application of computational fluid dynamics to three-dimensional modeling of the coronary artery and contrast flow by thrombolysis in the myocardial infarction (TIMI) frame count [9, 10]. Notably, these indices offer a convenient and wire-free approach, serving as an alternative to conventional hyperemic IMR for the assessment of coronary microvasculature in diverse clinical scenarios [11–15].

The aim of the present study was to assess the microvascular function in all coronary arteries of patients with different types of TTS and to compare angiography-derived IMR after recovery in a subset of patients. Furthermore, we sought to investigate a potential correlation of elevated IMR levels to in-hospital outcomes of patients with TTS.

## Methods

### Study design

This study is a retrospective, single center, age and gender matched analysis investigating coronary angiography-derived IMR (caIMR by FlashAngio, Rainmed Ltd., Suzhou, China) in a cohort of TTS patients and comparing it to non-culprit vessels of patients with ACS.

### Study population

All TTS patients were included in the International Takotsubo Registry (InterTAK Registry), established at the University Hospital of Zurich, that has already been described elsewhere [1]. All patients met the InterTAK Criteria for Takotsubo syndrome. In the present study, only patients enrolled at the University Hospital Zurich between 2009 and 2021 were included. TTS patients with coronary artery sclerosis with > 30% lumen narrowing were excluded.

A matched cohort of patients with ST-elevation myocardial infarction (STEMI) was selected from our institution’s ACS database (Zurich ACS Registry) to serve as the control group. This selection was based on previously published findings indicating that the microvascular function of non-culprit vessels remains independent of the acute event and microvascular dysfunction in the culprit vessel [16, 17]. Consequently, only non-culprit vessels without significant stenosis were considered for comparison. Non-culprit vessels that had previously served as culprit vessels in a past myocardial infarction or had undergone interventions such as percutaneous coronary intervention or percutaneous transluminal coronary angioplasty were excluded.

Furthermore, 10 TTS patients underwent coronary angiographies for various reasons, excluding acute coronary syndrome or recurrent TTS.

### Matching process

Patients with midventricular TTS were 1:1 matched according to age and sex with patients with apical TTS. All TTS patients were then further 1:1 matched with STEMI patients according to age and sex.

### CaIMR assessment

CaIMR was measured in all three epicardial vessels (left anterior descending artery [LAD], left circumflex artery [LCX], right coronary artery [RCA]) of TTS patients and in all vessels of ACS patients. Angiography derived fractional flow reserve and caIMR were obtained using angiographic views separated by at least 30° to reconstruct 3-D mesh reconstructions of the coronary arteries, as previously described using commercialized software (FlashAngio, Rainmed Ltd., Suzhou, China) [16, 18]. CaIMR was thereby calculated as: CaIMR = (Pd_hypap_*L)/(*K* * *V*_diastole_), where *L* represents the length from the inlet to the distal position of the target vessel, Pd_hypap_ is the mean pressure (unit: mmHg) at the distal position at maximal hyperemia which is proportional to the mean pressure during the diastole and thereby approximated by the FlashAngio software as the product of aortic pressure, and Angiography derived fractional flow reserve [18], *V*_diastole_ is the mean flow velocity (unit: mm/s) at the distal position at diastole derived from the thrombolysis in myocardial infarction (TIMI) frame count method and also automatically computed by the software [diastolic flow velocity = (contrast passing length)/(diastolic time interval)], and *K* is a constant (*K* = 2.1) to adjust the difference between resting and hyperemic flow velocity obtained from the literature [15, 19]. CaIMR elevation was defined according to vessel specific cut-offs (LAD: 22, LCX: 24 and RCA: 28), that have previously been published [20]. CaIMR were assessed by an operator (RF) blinded from the clinical data.

### Clinical endpoints

To assess the association between clinical outcomes and caIMR, an in-hospital composite endpoint consisting of death, cardiac arrest, ventricular arrhythmogenic events (documented ventricular tachycardia or fibrillation), and cardiogenic shock [21] was employed. Additionally, a TTS specific composite endpoint comprising death, ventricular thrombus, stroke, systemic embolism, TTS recurrence, and arrhythmogenic events (documented ventricular tachycardia or fibrillation) was used to assess the impact of caIMR on long-term outcomes.

### Ethics statement

The study was conducted according to the ethical principles of the Declaration of Helsinki. The local ethics committee reviewed the study protocol (BASEC-ID 2019-02402). Formal written consent is present for all patients prospectively enrolled in the InterTAK registry as well as in the control ACS group. For patients retrospectively included in the study before 2016 the relevant ethics committee partly waived the requirement to obtain informed consent.

### Statistical analysis

The distribution of variables was assessed using the Shapiro–Wilks test and histograms. Accordingly, continuous variables were described as mean ± standard deviation (SD) or median with interquartile range (IQR) and statistical significance was tested with Student *t*-test or Mann–Whitney *U* test as adequate. Categorical variables were reported as frequencies and percentages and analysed using Pearson χ^2^ test or Fisher exact test. CaIMR was compared using the Mann–Whitney *U* test. Multiple group comparisons were performed with the Kruskal–Wallis Test when comparing the three coronary arteries and the Dunn Test for consecutive pair-wise vessel comparison. Longitudinal caIMR measurements were compared with the paired *t*-test. Binary logistic regression analysis was performed to evaluate caIMR correlation with the clinical composite endpoint. A cox regression model was fitted to analyze the impact of elevated caIMR on the long-term composite endpoint. A two-sided *P*-value ≤ 0.05 was considered statistically significant. R version 4.2 (R Foundation, Vienna, Austria) was used for the statistical analyses. R version 4.2 as well as PRISM 9 (GraphPad, La Jolla, CA) were used for the compilation of graphs.

## Results

### Baseline clinical characteristics

A total of 192 patients (96 with TTS and 96 matched patients with ACS) were enrolled. CaIMR was assessable in 271 vessels of 96 patients with TTS: 137 (50.6%) in 48 patients with midventricular TTS and 134 (49.4%) in 48 patients with apical TTS. In the control group, caIMR was assessable in 170 non-culprit vessels of 96 patients. Within both the TTS and control groups, there were 12 (12.5%) male participants. The average age was 67.5 (± 11.5) years in the TTS group and 66.4 (± 11.4) years in the control group. The burden of cardiovascular risk factors was comparable in both groups. Baseline characteristics are reported in Table 1.

**Table 1:** Patient characteristics

Baseline	ACS	TTS	*p*-Value
	*N* = 96	*N* = 96	
Age, mean (SD)	66.4 (11.4)	67.5 (11.5)	0.496
Female sex, *N* (%)	84 (87.5)	84 (87.5)	1.000
Takotsubo type, *N* (%)			
Apical	NA	48 (50.0)	
Midventricular	NA	48 (50.0)	
Hypertension, *N* (%)	37 (38.9)	50 (52.1)	0.093
Diabetes mellitus, *N* (%)	12 (12.6)	15 (15.6)	0.699
Hypercholesterolemia, *N* (%)	30 (31.6)	27 (28.1)	0.497
LVEDP, mean of mmHg (SD)	20.16 (7.50)	20.37 (7.00)	0.902
LVEF, mean of % (SD)	49.54 (10.1)	41.17 (10.89)	< 0.001

*ACS* acute coronary syndrome; *LVEDP* left ventricular enddiastolic pressure; *LVEF* left ventricular ejection fraction; *NA* not applicable; *TTS* Takotsubo syndrome

### TTS vs ACS

**Fig. 1:**
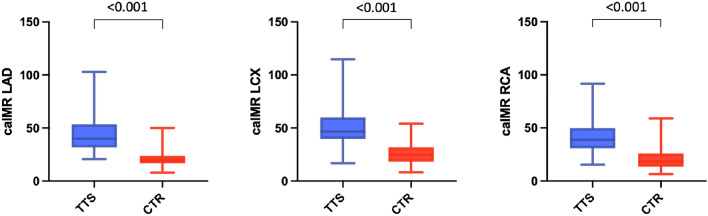
CaIMR in all three coronary arteries of our TTS patients was significantly higher compared to the respective vessels of the control group

Patients with TTS presented with elevated caIMR in at least one coronary artery in 100%, in at least two coronary arteries in 97% and in three coronary arteries in 68%. In patients with STEMI elevated caIMR was present in 53% of non-culprit vessels (*p* < 0.001). CaIMR in each of the three coronary arteries of the TTS cohort was significantly higher compared to the respective control vessels of the control group (LAD: caIMR during TTS 39.9 [31.7–52.2] vs 19.8 [17.3–23.4] in the control group, *p* < 0.001; LCX: 46.7 [40.4–59.9] vs 24.7 [18.4–31.6], *p* < 0.001; RCA: 38.8 [31.0–49.5] vs 18.4 [13.8–26.0], *p* < 0.001) (Fig. 1). Comparing all three main vessels in the TTS cohort, a significant difference could be detected regarding median caIMR (39.9 [LAD] vs 46.7 [LCX] vs 38.8 [RCA], *p* < 0.001), with highest caIMR levels in the LCX (LCX vs LAD: *p* = 0.009; and LCX vs RCA: *p* < 0.001).

### Apical vs midventricular TTS

**Fig. 2:**
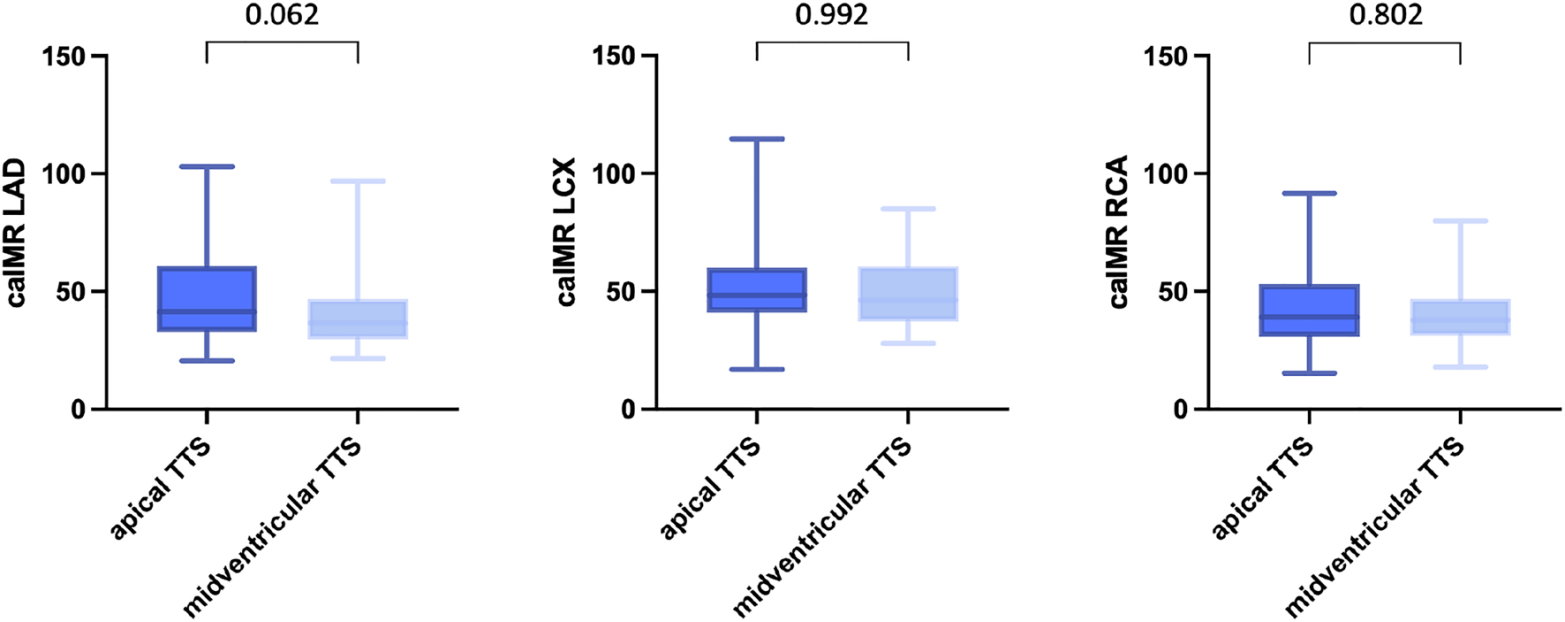
There was no significant difference in caIMR between the apical and the midventricular type of TTS in any of the three main coronary arteries

There was no significant difference in caIMR between the apical and the midventricular type of TTS in any of the three main coronary arteries. However, a trend towards higher caIMR in the LAD in patients with an apical form of TTS compared to patients with midventricular TTS was observed (LAD: 41.3 [32.9–60.5] vs 36.6 [29.9–46.7], *p* = 0.062; LCX: 48.4 [41.2–59.4] vs 46.3 [38.4–59.9], *p* = 0.992; RCA: 39.1 [30.8–52.9] vs 37.8 [31.3–46.7], *p* = 0.802; Fig. 2).

### Longitudinal CaIMR analysis

**Fig. 3:**
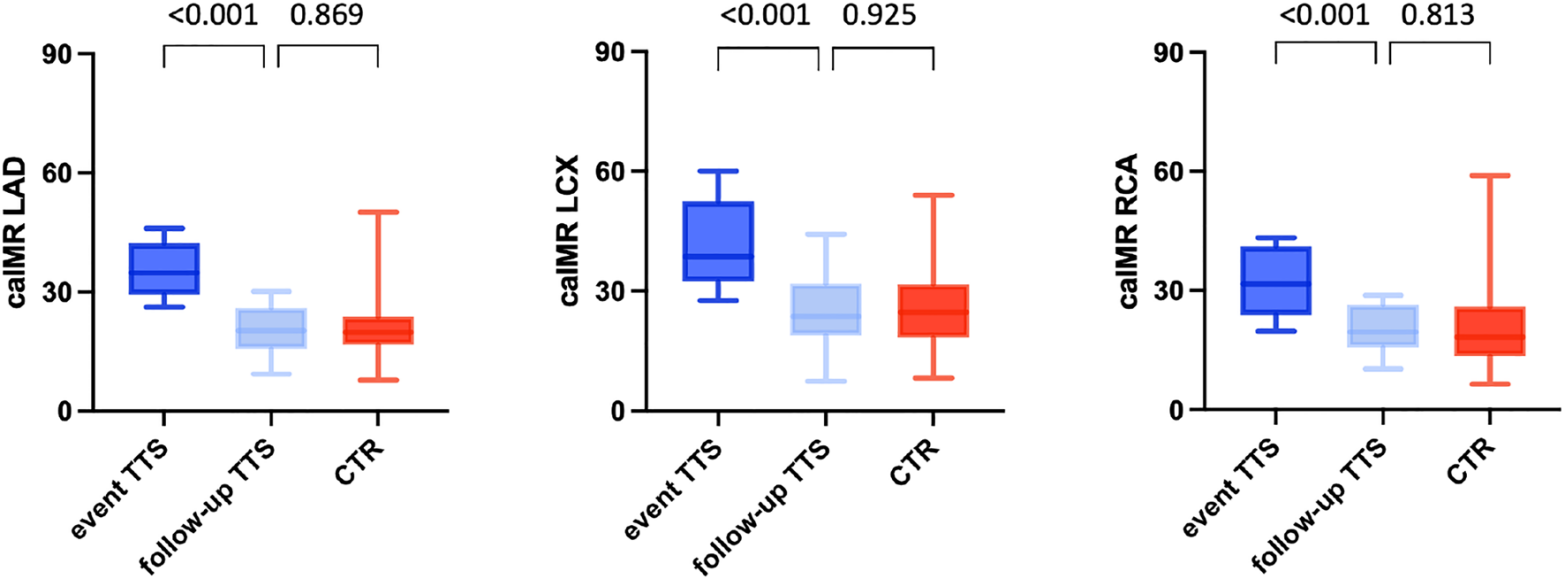
CaIMR normalized in all TTS patients at the time of follow-up. Comparing these TTS patients` follow up caIMR with the caIMR of our normal control vessels, no significant difference could be observed

Of 96 TTS patients, 16 patients (16.7%) had follow-up angiographies. Two patients (2.1%) had recurrent TTS and four patients (4.2%) had ACS and were therefore excluded from the analysis. The remaining 10 patients where eligible for analysis. These reference exams were median 28 months (IQR 20–35) apart from the index angiography (during acute TTS). In two patients the RCA could not be evaluated due to technical aspects of the coronary angiography both at the time of the TTS event and at follow-up. However, caIMR normalized in all patients at follow-up (LAD: median caIMR during TTS event 34.8 [29.9–41.1] vs 20.3 [16.0–25.3] at follow-up, *p* < 0.001; LCX: 38.7 [32.9–50.1] vs 23.7 [19.4–30.5], *p* < 0.001; RCA: 31.7 [25.0–39.1] vs 19.6 [17.1–24.0], *p* < 0.001; Fig. 3). Comparing the TTS patients’ follow-up caIMR with the caIMR of non-culprit ACS vessels, no significant difference could be observed (LAD: median caIMR at TTS follow-up 20.3 [16.0–23.3] vs 19.8 [17.3–23.4] in the control group, *p* = 0.869; LCX: 23.7 [19.4–30.5] vs 24.7 [18.4–31.6], *p* = 0.925; RCA: 19.6 [17.1–24.0] vs 18.4 [13.8–26.0]; *p* = 0.813; Fig. 3).

### In-hospital outcomes

Neither left ventricular end diastolic pressure nor LVEF nor Troponin levels significantly correlated with caIMR (Suppl. Figures 1, 2 and 3).

**Fig. 4:**
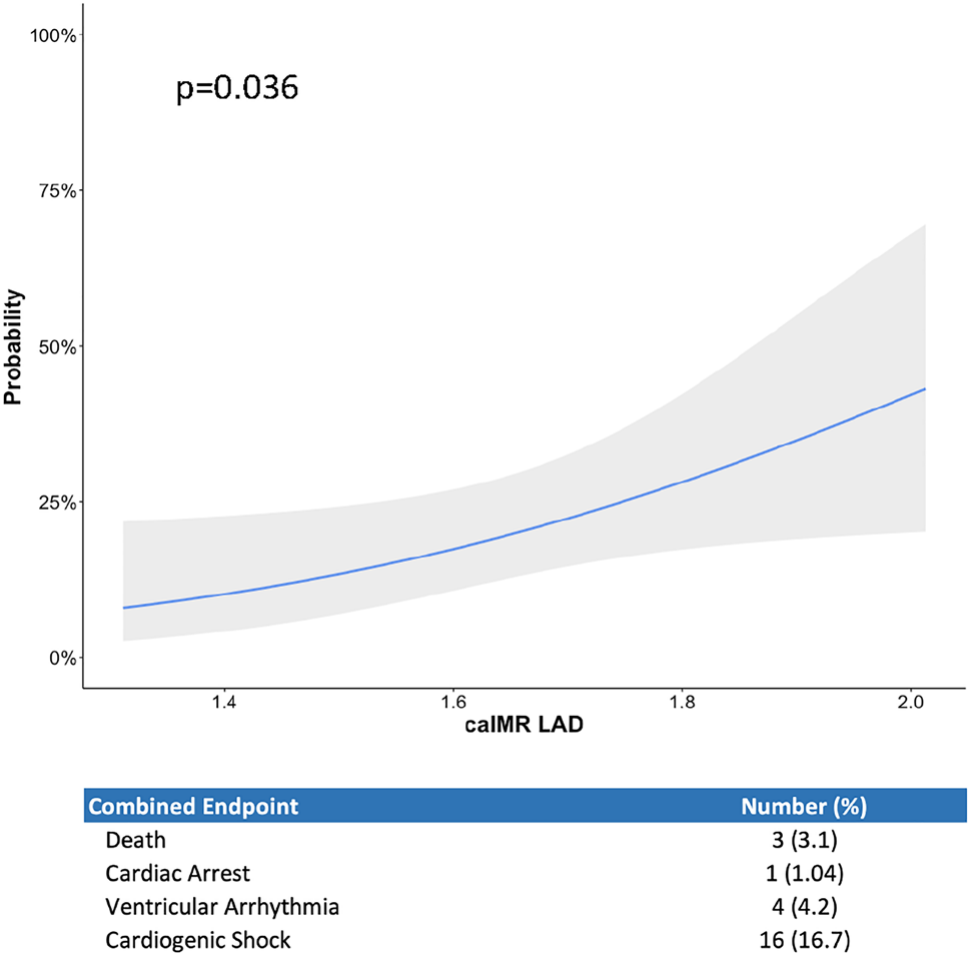
Logarithmized (10log) caIMR of patients correlates with the probability of in-hospital MACE consisting of death, cardiac arrest, ventricular arrythmia, and cardiogenic shock

However, the extent of caIMR elevation in the LAD during the acute event significantly correlated with the TTS-specific in-hospital composite endpoint of death, cardiac arrest, ventricular arrythmia and cardiogenic shock under vasopressors (19.8% of patients, *p* = 0.036; Fig. 4).

### Long-term outcomes

In univariate cox-regression analyses, there were no significant associations observed between logarithmized caIMR values in the LAD, LCX or RCA and the 1 year long-term composite endpoint comprising death, ventricular thrombus, stroke, systemic embolism, TTS recurrence, and arrhythmogenic events (LAD: HR: 1.73 [95% CI: 0.15–20] *p* = 0.662; LCX: HR: 0.69 [95%CI: 0.04–13], *p* = 0.804; RCA: HR: 1.73 [95%CI: 0.02–4.2], *p* = 0.334).

## Discussion

In the present study, the clinical implications of elevated IMR in patients with TTS were investigated. The main results of the investigation are: (I) elevated caIMR could be consistently demonstrated in the TTS cohort; (II) apical and midventricular TTS form showed comparable microvascular dysfunction patterns, only a trend towards higher caIMR in the LAD of the apical types was observed; (III) impairment of the microvascular function in TTS showed a transient nature with complete recovery at a median follow-up of 28 months; and (IV) in-hospital outcomes correlated significantly with the extent of caIMR elevation at the index coronary angiography.

Dysfunctional coronary microcirculation in the acute TTS phase has previously been described. In a small cohort of TTS patients, Kim et al. reported that invasively measured IMR was similarly impaired as compared to STEMI patients after revascularization [22]. In contrast to the microvascular dysfunction following a reperfused STEMI [23, 24], impaired microcirculatory function in TTS is likely due to excessive vasoconstriction and augmented sympathetic responses to acute stress [22, 25] and subsequent catecholamine excess [4, 26–28]. High catecholamine levels following activation of the sympathetic nervous system have been associated with the development of endothelial CMD either via direct cardiomyocyte toxicity or by inducing coronary artery spasms (evidence of decreased plasma levels of microRNA 125a-5p and increased levels of its target endothelin-1) resulting in inadequate myocardial blood supply [29–31].

Angiography-derived IMR, such as caIMR, has been developed as a non-invasive alternative to wire-based IMR and is nowadays a validated and useful tool to assess coronary microcirculatory function [15, 16]. Since no wire introduction into the coronary arteries is required for this technique, angiography-derived IMR is a safe and feasible alternative to wire-based approaches for the evaluation of the three main epicardial vessels in patients with TTS.

The present study was able to demonstrate higher caIMR in the acute setting of TTS compared to non-culprit vessels of matched ACS patients, which have been proven to function as a control and therefore represent a valid model in wire-based IMR studies [16, 17]. In addition, it was demonstrated that caIMR elevation is not limited to one specific coronary artery but is present in the three main coronary arteries with highest caIMR in the LCX followed by the LAD and the RCA. These findings are in contrast with previous evidence reporting higher caIMR in the LAD of TTS patients [9]. A possible explanation for the discrepancy in the magnitude of the differences between caIMR in the LAD and LCX is the relatively higher number of TTS patients with midventricular types in our cohort.

By comparing TTS patients to a matched control group, the presence of elevated caIMR in the three main epicardial vessels was also endorsed. In a comparison of all vessels (all vessels of TTS patients and all culprit + non-culprit vessels of STEMI patients), these findings were consistent (Suppl. Material Page 1). This could possibly be of help in differentiating ACS from TTS with bystander coronary artery disease when the cause of an apical wall motion abnormality is in doubt, but due to a high prevalence of elevated caIMR in non-culprit ACS vessels (53%) this hypothesis will need evaluation in a prospective all-comer cohort. However, the high percentage of elevated caIMR in non-culprit vessels of ACS patients in this study is in contrast to previously published results but could be a consequence of the unusually high female prevalence in our ACS cohort, which can be mainly attributed to the matched fashion of the study, as TTS cohorts usually have a female prevalence of ~ 85%.

We observed a trend towards higher caIMR values in the LAD of patients with apical TTS types compared to midventricular TTS types. This trend could potentially achieve statistical significance with an increased number of patients. While the elevated caIMR in the LAD could reflect the akinesia in the anterior wall and apical cap, IMR alone does not appear to fully explain the different wall motion patterns. In contrast to our findings, Sans-Rosello et al. demonstrated differences in caIMR between different TTS types. However, in their study, the authors were splitting apical and midventricular TTS types into “limited” and combined groups (“apical limited” vs “apical + midventricular” and “midventricular limited” vs “midventricular + basal”). When considering only the “limited” groups, their results seem comparable to the findings in this study.

This study includes 10 TTS patients with follow-up angiographies for various reasons (no recurrent TTS) at median of 28 months from the TTS event. A transient nature of elevated caIMR in TTS was demonstrated, independent of the extent of caIMR elevation during the acute TTS event. Therefore, the study provides evidence that patients who suffer TTS do not necessarily have a certain degree of chronic CMD at baseline but can resemble the general population apart from their TTS event. These findings support those reported by Rivero and colleagues, who demonstrated an inverse relation between invasively measured IMR and the duration between the time of symptom onset and hospital admission in a small TTS cohort of 15 patients, suggesting a transient nature of caIMR elevation in TTS [32].

In this cohort, no statistically significant correlation between cardiac troponins, LVEF or LVEDP and caIMR was observed (Suppl. Figure 2). The lack of correlation with troponin is challenging to interpret, however, the prognostic role of troponin in TTS has not been fully elucidated and may be influenced by other factors. When considering wall-motion abnormalities, it is important to note that LVEF and, consequently, LVEDP during the acute event are influenced not only by the extent of akinesia but also by the degree of hyperkinesia, as seen in the basal myocardium in the apical TTS variant. Moreover, this correlation might vary significantly between typical and atypical TTS forms. While in another previously published study [9], the caIMR in the LAD correlated with LVEF, in our study, with a substantially higher number of atypical TTS cases, these findings could not be reproduced.

Since from a physiological standpoint, an increased LVEDP might correspond to elevated microcirculatory resistance, a sub-analysis including only patients with normal LVEDP (≤ 12 mmHg) at the time of angiography was performed. In this context, comparable results to the main analysis were obtained (Suppl. Material p. 1).

Similar to the prognostic impact of elevated caIMR in ACS patients [15, 16], this study demonstrated that the extent of caIMR elevation might predict in-hospital outcomes in patients with TTS. While in ACS cohorts CMD most likely correlates with infarct size and myocardial scaring, thereby influencing long-term outcomes, the transient nature of caIMR elevations in TTS patients might mainly influence the in-hospital outcome. In this regard, the TTS-specific major in-hospital adverse cardiac event (MACE) rate of 19.8% defined as death, cardiac arrest, ventricular arrhythmia and cardiogenic shock as a result of cardiogenic shock was associated with higher caIMR values in the LAD. The implications on in-hospital outcomes, together with the evidence for the transient nature of elevated caIMR in TTS suggest that the microvascular function might represent a promising therapeutic target in the acute TTS event.

Sans-Roselló et al. recently published their cohorts’ one-year follow-up MACE rate of 21.2% in 166 TTS patients in which long-term outcome significantly correlated with the extent of caIMR elevation at the time of the acute TTS event [33]. Of note, the end-point definition included all-cause death, cardiovascular death, heart failure event, acute myocardial infarction and symptomatic tachyarrhythmia/bradyarrhythmia, which occurred also after complete recovery and, therefore, might not be a consequence of the TTS event, but rather the old age and high rate of comorbidities in patients with TTS. However, within the cohort examined in this study, increased caIMR did not exhibit a significant association with established TTS-specific adverse long-term outcomes. This finding aligns with our initial expectations, indicating that elevated caIMR may not be a major contributor to established TTS-specific adverse long-term outcomes but rather in-hospital outcomes.

### Limitations

The design of the present study did not allow for comparison of caIMR to invasively measured IMR. However, reports validating the caIMR in comparison to invasive hyperemic IMR have been published [13, 16]. The number of TTS patients with follow-up angiographies in our study was limited and the timing of the follow-up exam varied from 22 to 35 months. Therefore, no conclusions can be drawn on the exact timely dynamics of caIMR elevations and its recovery in the setting of TTS.

## Conclusion

CaIMR represents a valid wire- and hyperemic agent-free alternative to invasively measure microvascular resistance in the three main coronary arteries of patients with TTS. Like the impairment of the global cardiac function, elevated caIMR appeared to be transient in TTS with high caIMR during the acute setting in the three main coronary arteries and normalization at follow-up. The extent of caIMR elevation correlated with in-hospital TTS-specific MACE. The transience of caIMR elevation, together with its implications on in-hospital outcomes, suggest that the impaired microvascular function might represent a promising therapeutic target in the acute TTS setting.

## Supplementary Information

Below is the link to the electronic supplementary material.

Supplementary file1 (PDF 81 KB)

## Abbreviations

ACS: Acute coronary syndrome
(ca)IMR: (Coronary angiography derived) index of microcirculatory resistance
CMD: Coronary microvascular dysfunction
LAD: Left anterior descending artery
RCA: Right coronary artery
LCX: Ramus circumflexus
LVEDP: Left ventricular enddiastolic pressure
LVEF: Left ventricular ejection fraction
STEMI: ST-elevation myocardial infarction
TTS: Takotsubo syndrome
