# Supplementary material for: Coronary microvascular dysfunction in Takotsubo syndrome: an analysis using angiography-derived index of microcirculatory resistance
Source: Clin Res Cardiol. 2023 Nov 20;113(12):1629–37. doi: 10.1007/s00392-023-02329-7 (PMC11579140; doi:10.1007/s00392-023-02329-7)
Supplement: Supplementary file 1 — Supplementary file1 (PDF 81 KB) [file 392_2023_2329_MOESM1_ESM.pdf]

Supplementary Material

TTS vs ACS, patients with normal LVEDP

Considering only patients with an LVEDP ≤ 12mmHg (N=11 in the TTS and N=11 in the control group), caIMR in each of the three coronary arteries of the TTS cohort was significantly higher compared to the respective control vessels of the control group (LAD: caIMR in the TTS 50.2 [34.1 – 79.3] vs. 19.23 [18.2 – 19.4] in the control group, p<0.001; LCX: 61.4 [55.4 – 77.4] vs. 18.3 [12.3 – 26.9], p<0.001; RCA: 43.9 [38.0 – 52.2] vs. 26.1 [13.1 – 32.0], p<0.001).

TTS vs ACS, all vessels

Considering all vessels (all three coronary arteries of TTS patients and culprit + non-culprit vessels of STEMI patients) of all included patients, caIMR in each of the three coronary arteries of the TTS cohort was still significantly higher compared to the control group (LAD: caIMR during TTS 39.9 [31.7 – 52.2] vs. 24.3 [18.6 – 40.3] in the control group, p<0.001; LCX: 46.7 [40.4 – 59.9] vs. 25.7 [19.0 – 35.5], p<0.001; RCA: 38.8 [31.0 – 49.5] vs. 23.5 [17.2 – 38.9], p<0.001).

Supplementary Figures

Suppl. Fig 1.

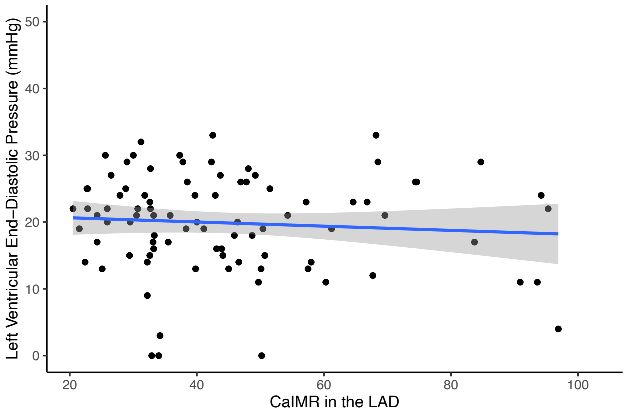

Suppl. figure 1: Correlation of caIMR with LVEDP.

Suppl. Fig 2.

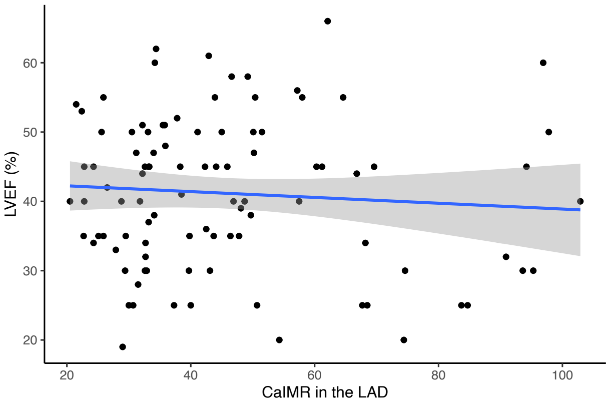

Suppl. figure 2: Correlation of caIMR with LVEF.

Suppl. Fig 3.

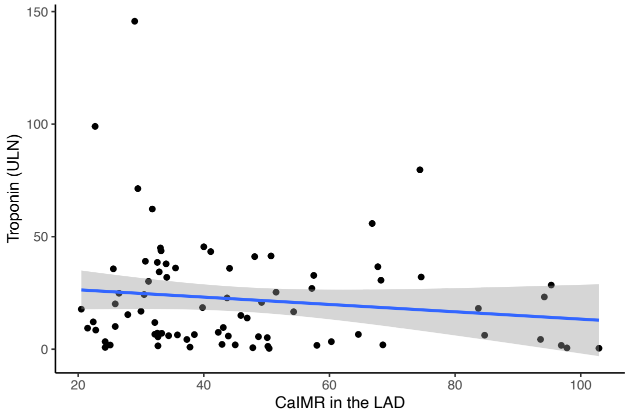

Suppl. figure 3: Correlation of caIMR with Troponin levels.
